# Salicortin-Derivatives from *Salix pseudo-lasiogyne* Twigs Inhibit Adipogenesis in 3T3-L1 Cells via Modulation of C/EBPα and SREBP1c Dependent Pathway

**DOI:** 10.3390/molecules180910484

**Published:** 2013-08-30

**Authors:** Mina Lee, Sang Hoon Lee, Jimmy Kang, Heejung Yang, Eun Ju Jeong, Hong Pyo Kim, Young Choong Kim, Sang Hyun Sung

**Affiliations:** 1 College of Pharmacy and Research Institute of Pharmaceutical Science, Seoul National University, Daehak-Dong, Gwanak-Gu, Seoul 151-742, Korea; 2 Department of Agronomy and Medicinal Plant Resources, College of Life Sciences and Natural Resources, Gyeongnam National University of Science and Technology, Jinju 660-758, Korea; 3 College of Pharmacy, Ajou University, Suwon 443-749, Korea

**Keywords:** *Salix pseudo-lasiogyne*, Salicaceae, 3T3-L1, adipogenesis, adipocyte differentiation, C/EBPα, SREBP1c, obesity

## Abstract

Obesity is reported to be associated with excessive growth of adipocyte mass tissue as a result of increases in the number and size of adipocytes differentiated from preadipocytes. To search for anti-adipogenic phytochemicals, we screened for inhibitory activities of various plant sources on adipocyte differentiation in 3T3-L1 preadipocytes. Among the sources, a methanolic extract of *Salix pseudo-lasiogyne* twigs (Salicaceae) reduced lipid accumulation in a concentration-dependent manner. During our search for anti-adipogenic constituents from *S*. *pseudo-lasiogyne*, five salicortin derivatives isolated from an EtOAc fraction of this plant and bearing 1-hydroxy-6-oxo-2-cyclohexene-carboxylate moieties, namely 2′,6′-*O*-acetylsalicortin (**1**), 2′-*O*-acetylsalicortin (**2**), 3′-*O*-acetylsalicortin (**3**), 6′-*O*-acetylsalicortin (**4**), and salicortin (**5**), were found to significantly inhibit adipocyte differentiation in 3T3-L1 cells. In particular, 2′,6′-*O*-acetylsalicortin (**1**) had the most potent inhibitory activity on adipocyte differentiation, with an IC_50_ value of 11.6 μM, and it significantly down-regulated the expressions of CCAAT/enhancer binding protein α (C/EBPα) and sterol regulatory element binding protein 1 (SREBP1c). Furthermore, 2′,6′-*O*-acetylsalicortin (**1**) suppressed mRNA expression levels of C/EBPβ during the early stage of adipocyte differentiation and stearoyl coenzyme A desaturase 1 (SCD-1), acetyl-CoA carboxylase (ACC), and fatty acid synthase (FAS) expression, target genes of SREBP1c. In the present study, we demonstrate that the anti-adipogenesis mechanism of 2′,6′-*O*-acetylsalicortin (**1**) may be mediated via down-regulation of C/EBPα and SREBP1c dependent pathways. Through their anti-adipogenic activity, salicortin derivatives may be potential novel therapeutic agents against obesity.

## 1. Introduction

Obesity is a condition resulting from the excessive storage of fat in the body. The prevalence of obesity is dramatically increasing and, in many industrialized countries, has become a health risk because of the high correlation between obesity and metabolic disorders such as type 2 diabetes, cardiovascular disease, hyperlipidemia, and hypertension [1,2]. Despite much progress in obesity research over the past decades, anti-obesity medicines have not been easy to develop because of the complexity of obesity and its association with other metabolic disorders. Current remedies provided by the diet industry have failed to result in long-term maintenance of weight loss in obese patients and adverse effects of some remedies have been reported [3,4]. Therefore, novel therapeutic strategies are needed to treat obesity.

Adipose tissue growth involves formation of new adipocytes from precursor cells leading to an increase in both the number and size of adipocytes [5,6]. Regulation both size and number of adipocytes may provide a novel therapeutic approach to the treatment of obesity [7].

*Salix* species have been used in the treatment of fever, pain, and inflammation since ancient times [8,9]. Moreover, *Salix* species have also been reported to have anti-inflammatory [10], anti-oxidant [11], anti-tumor [12], and anti-obesity [13] effects. Recently, it was reported that *Salix pseudo-lasiogyne* H. Lev., used for the treatment of pain and fever in Korean traditional medicine, has a cognitive-enhancing effect [14]. During a search for anti-adipogenic phytochemicals from medicinal plants, we found five salicortin-derivatives isolated from *S. pseudo-lasiogyne* that can significantly inhibit adipocyte differentiation in 3T3-L1 cells. To date, there are no known reports on the anti-adipogenic activity of *S*. *pseudo-lasiogyne* in 3T3-L1 cells and the mechanism underlying such effects of salicortin derivatives is unknown. In the present study, we elucidate the anti-adipogenic mechanism of salicortin derivatives isolated from *S*. *pseudo-lasiogyne*.

## 2. Results and Discussion

### 2.1. Results

**Figure 1:**
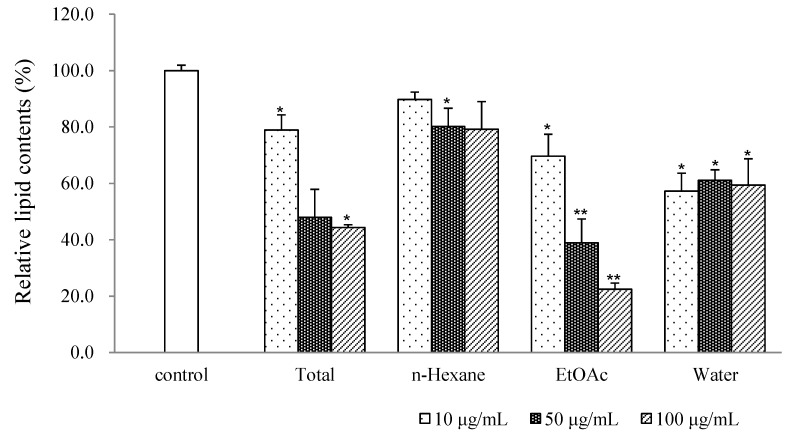
Effects of the methanolic extract and fractions of *Salix pseudo-lasiogyne* on adipocyte differentiation in 3T3-L1 cells.

**Figure 2:**
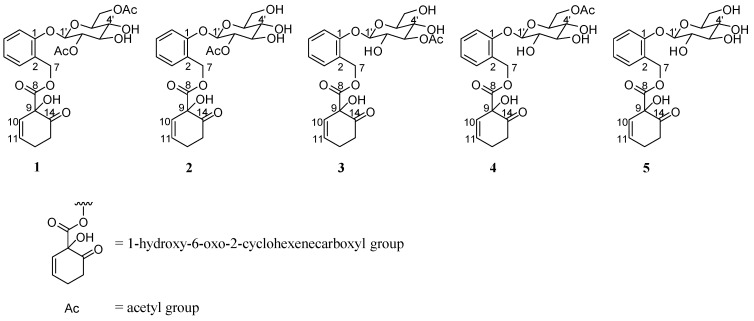
Structures of salicortin-derivatives isolated from *Salix pseudo-lasiogyne*.

During screening to determine the anti-adipogenic activity of natural products, we observed that a methanolic extract of *S. pseudo-lasiogyne* twigs exhibited inhibitory activity on adipocyte differentiation in 3T3-L1 preadipocytes (Figure 1). The methanolic extract of *S*. *pseudo-lasiogyne* twigs was successively fractioned into *n*-hexane, EtOAc, and H_2_O fractions. Among those fractions, the EtOAc fraction inhibited adipocyte differentiation in a concentration-dependent manner (10 to 100 μg/mL) and yielded five salicortin derivatives, identified as 2′,6′-*O*-acetylsalicortin (**1**), 2′-*O*-acetylsalicortin (**2**), 3′-*O*-acetylsalicortin (**3**), 6′-*O*-acetylsalicortin (**4**), and salicortin (**5**) (Figure 2). These compounds are composed of 1-hydroxy-6-oxocyclohex-2-enecarboxylate, catechol, *β*-D-glucose, and an acetyl group.

3T3-L1 preadipocytes were treated with various concentrations of the methanolic extract and fractions of *Salix pseudo-lasiogyne* during differentiation (day 0–8). On day 8, cultures were stained with Oil Red O and the lipid contents were quantified spectrophotometrically at 544 nm. Relative lipid contents (%) were calculated as 100 × [(absorbance of sample-treated – absorbance of undifferentiated control)/(absorbance of differentiated control – absorbance of undifferentiated control)]. Results represent the mean ± SD of three independent experiments, each performed using triplicate wells. * *p* < 0.05, ** *p* < 0.01, *** *p* < 0.001, compared with the differentiated cells.

**Figure 3:**
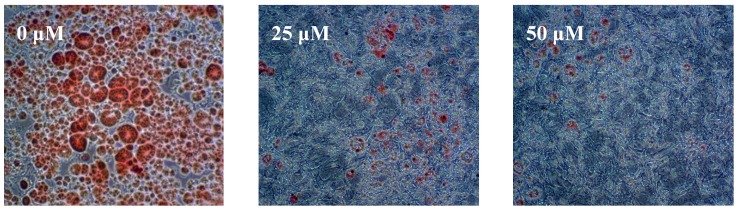
Effects of 2′,6′-*O*-acetylsalicortin (**1**) on adipocyte differentiation in 3T3-L1 cells.

To examine the effect of salicortin derivatives **1**–**5**, isolated from *S*. *pseudo-lasiogyne* on the differentiation of preadipocytes to adipocytes, confluent 3T3-L1 preadipocytes were treated with compounds at various concentrations during differentiation (days 0–8). On day 8, the lipid contents, stained with Oil Red O (ORO), were photographed via a phase-contrast microscope and quantified spectrophotometrically at 544 nm. Of the compounds tested in our assay system, 2′,6′-*O*-acetylsalicortin (**1**) had the most potent inhibitory activity on adipocyte differentiation in 3T3-L1 cells, with an IC_50_ value of 11.6 μM, compared to the activity of the positive control epigallocatechin-3-gallate (EGCG) (Table 1) [15]. As shown in Figure 3, treatment with 2′,6′-*O*-acetylsalicortin (**1**) decreased the lipid content at concentrations of 25 μM and 50 μM, compared to the content of fully differentiated adipocyte. Compounds **2**–**4**, with one acetyl group in the β-D-glucose moiety, inhibited adipocyte differentiation less than that of 2′,6′-*O*-acetylsalicortin (**1**) and had similar IC_50_ values (24.6 μM, 25.0 μM, and 27.1 μM, respectively). In comparison, compound **5**, without an acetyl group in β-D-glucose, showed the least suppressive effect among the salicortin derivatives and had an IC_50_ value of 53.5 μM (Table 1).

**Table 1:** Effects of the salicortin-derivatives isolated from *Salix pseudo-lasiogyne* on adipocyte differentiation in 3T3-L1 preadipocytes.

Compound	IC_50_ (μM)
**1**	11.6
**2**	24.6
**3**	25.0
**4**	27.1
**5**	53.5
**EGCG**	112.0

IC_50_ means the 50% inhibitory concentration (μM) on on adipocyte differentiation in 3T3-L1 preadipocytes. EGCG was used as the positive control.

Confluent 3T3-L1 preadipocytes were differentiated into adipocytes in a medium with different concentrations (0, 25, and 50 μM) of 2′,6′-*O*-acetylsalicortin (**1**). On day 8, stained cells with Oil Red O were photographed with a phase-contrast microscope (original magnification ×100).

**Figure 4:**
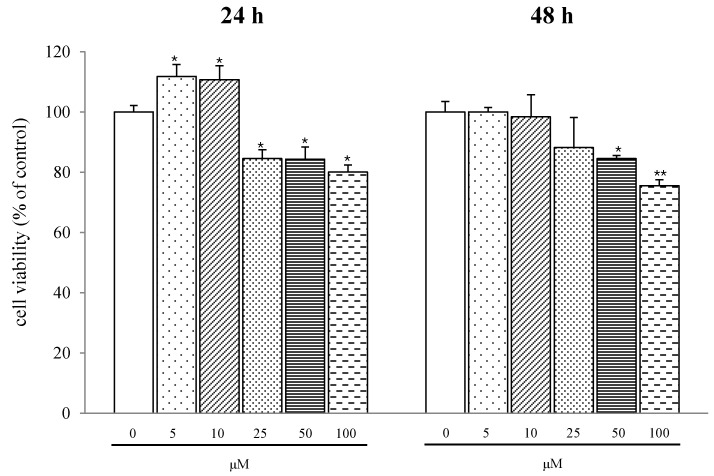
Effect of 2′,6′-*O*-acetylsalicortin (**1**) on 3T3-L1 preadipocyte proliferation.

**Figure 5:**
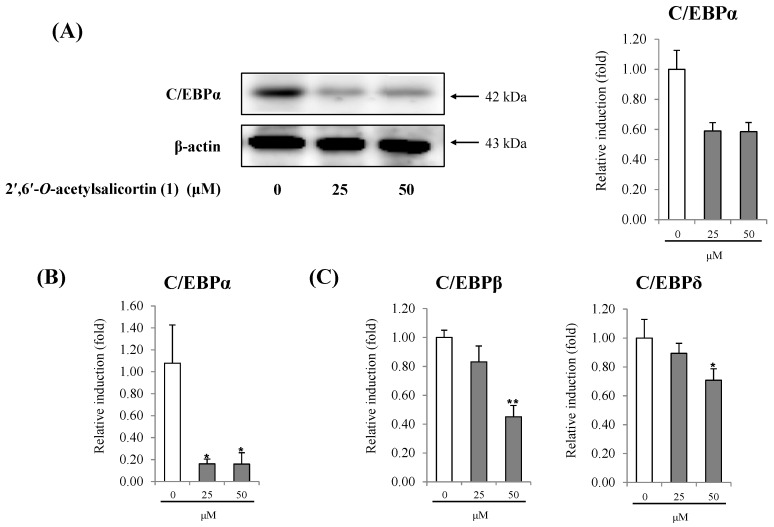
Effect of 2′,6′-*O*-acetylsalicortin (**1**) on anti-adipogenesis mediated by the down-regulation of C/EBPα dependent pathways.

**Figure 6:**
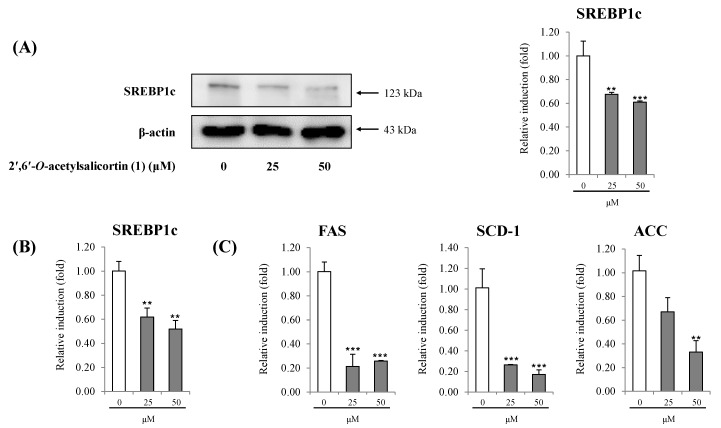
Effect of 2′,6′-*O*-acetylsalicortin (**1**) on anti-adipogenesis mediated by the down-regulation of SREBP1c dependent pathways.

Among the five adipocyte differentiation-suppressing salicortin derivatives, further investigation of **1**, the most potent compound from *S*. *pseudo-lasiogyne*, was carried out to reveal its anti-adipogenic activity on 3T3-L1 cells. Compound **1** slightly decreased the proliferation of preadipocytes in a concentration-dependent (range of 25 μM to 100 μM) manner at 24 h and 48 h (Figure 4). A series of programmed gene expression changes that occur in adipokines affect adipocyte differentiation. Therefore, we further investigated the anti-adipogenic mechanism of **1** by using quantitative real-time PCR and/or western blot analyses to determine the expression of adipogenic transcription factor at concentrations of **1** of 25 μM and 50 μM. Treatment with **1** markedly lowered the regulation of C/EBPα, compared to that from fully differentiated adipocytes. In addition, treatment with **1** reduced gene expression levels of C/EBPβ and C/EBPδ, which are activated in the initial stage of adipogenesis in preadipocytes (Figure 5). Moreover, treatment with **1** suppressed the protein expression level of SREBP1c in a concentration-dependent manner (Figure 6A). Similarly, the gene expression level of SREBP1c in cells treated with **1** also decreased (Figure 6B). The decreases in gene expression levels of SCD-1, FAS, and ACC (Figure 6C), the target genes of SREBP1c, by **1** during adipocyte differentiation imply that the inhibition of adipocyte differentiation is mediated by the regulation of lipogenesis [16,17].

3T3-L1 preadipocytes were incubated with different concentrations of 2′,6′-*O*-acetylsalicortin (**1**) (5, 10, 25, 50, and 100 μM) for 24 and 48 h. Cell proliferation was determined by MTT assay. MTT assay was quantified by spectrophotometrically at 540 nm. Results represent the mean ± SD of three independent experiments, each performed using triplicate wells. * *p* < 0.05, ** *p* < 0.01, compared with the control.

3T3-L1 preadipocytes were untreated, as a control, of treated with different concentrations of 2′,6′-*O*-acetylsalicortin (**1**) (25 and 50 μM) during adipocyte differentiation. (**A**), (**B**) On day 8, gene and protein expression levels of C/EBPα were estimated by western blot and quantitative real-time RT-PCR analysis analysis, respectively. (**C**) On day 2, gene and protein expression levels of C/EBPβ and C/EBPδ, which rise early induced to differentiate in preadipocyte were examined by quantitative real-time RT-PCR analysis. Results represent the mean ± SD of three independent experiments, each performed using triplicate wells. * *p* < 0.05, ** *p* < 0.01, *** *p* < 0.001, compared with the differentiated cells.

3T3-L1 preadipocytes were untreated, as a control, of treated with different concentrations of 2′,6′-*O*-acetylsalicortin (**1**) (25 and 50 μM) during adipocyte differentiation. On day 8, total RNA and total protein were harvested from adipocyte differentiation. (**A**), (**B**) On day 8, gene and protein expression levels of SREBP1c were estimated by western blot and quantitative real-time RT-PCR analysis analysis, respectively. (**C**) Gene expression levels of SCD-1, FAS, and ACC, the target genes of SREBP1c, were quantified by quantitative real-time RT-PCR analysis. Results represent the mean ± SD of three independent experiments, each performed using triplicate wells. * *p* < 0.05, ** *p* < 0.01, *** *p* < 0.001, compared with the differentiated cells.

### 2.2. Discussion

Obesity is a condition associated with excessive growth of adipose tissue resulting from an increase in the number and size of adipocytes differentiated from preadipocytes [6,18]. Not in order. Therefore, suppression of preadipocyte proliferation and their differentiation to adipocytes, thereby reducing the genesis and development of adipose tissue, may be useful as an anti-obesity method. On that basis, we investigated therapeutic anti-obesity agents in 3T3-L1 cells by assessing their effects on adipocyte differentiation. Previous studies have reported that phytochemicals isolated from medicinal plants can inhibit adipocyte differentiation in 3T3-L1 preadipocytes. For example, EGCG, isolated from green tea, suppresses adipogenesis by induction of apoptosis in preadipocytes, down-regulation of adipokines, and activation of the AMPK pathway. During a search for naturally occurring anti-adipogenic products from medicinal plants, we observed anti-adipogenic activity of salicortin derivatives isolated from *S*. *pseudo-lasiogyne* twigs in 3T3-L1 cells.

The phytochemical salicortin is abundant in poplar and willow bark, and its presence has been reported throughout the Salicaceae family [19]. Pharmacological studies of salicortin isolated from such plants have shown it to have anti-cancer activity [20]. Its anti-adipogenic effect was suggested by others who reported that a salicortin-rich *Populus balsamifera* extract inhibited adipocyte differentiation in 3T3-L1 cells [19]. Salicortin is a phenolic glycoside bearing a 1-hydroxy-6-oxo-2-cyclohexenecarboxylate moiety, a structure related to the development of aspirin [21]. In addition, salicortin derivatives bearing the 1-hydroxy-6-oxo-2-cyclohexenecarboxylate moiety significantly inhibit lipopolysaccharide-induced nitric oxide production in BV2 microglial cells than other compounds isolated from *S*. *pseudo-lasiogyne* [14]. In addition, compounds with a 1-hydroxy-6-oxo-2-cyclohexenecarboxylate moiety have exhibited potent inhibitory activities on adipocyte differentiation [22]. In present study, compounds **1**–**5**, identified as salicortin derivatives, significantly inhibited adipocyte differentiation in 3T3-L1 cells. These results enable analysis of the structure-activity relationship of these compounds. A portion of their anti-adipogenic activity may be attributed to 1-hydroxy-6-oxo-2-cyclohexenecarboxylate moiety, a bioactive degradation product of salicortin, but further investigation is needed to confirm the implication of this metabolite in the anti-adipogenic activity of salicortin derivatives. As shown in Table 1, the adipose differentiation IC_50_ values of the salicortin derivatives tested in this study suggest that the presence and numbers of acetyl groups in *β*-D-glucose can affect their anti-adipogenic activity. To determine accurately the structure-activity relationship, further study of the inhibitory effects on adipocyte differentiation by compounds with more structural variety is needed. In addition, a variety of compound structures including1-hydroxy-6-oxo-2-cyclohexenecarboxylate should be assessed to clarify the relationships between structure and activity. Regardless, our results suggest the importance of salicortin metabolites in the inhibition of adipocyte differentiation.

The protein C/EBPα is reported to be a key regulator of adipogenesis and directly affects the development of fat cells [6,23]. This transcription factor is reported to coordinate the expression of genes involved in initiating and maintaining adipocyte phenotype [24]. Two other members of the C/EBP family, C/EBPβ and C/EBPδ, are expressed earlier than C/EBPα during adipogenesis in 3T3-L1 cells and are responsible for regulating C/EBPα expression [23]. The messenger protein cAMP, an inducer of adipogenesis *in vitro* and a component of most pro-differentiative regimens, can enhance expression of both C/EBPα and C/EBPβ; subsequently, C/EBPβ and glucocorticoids can increase the induction of C/EBPδ [25,26]. Early C/EBPβ and C/EBPδ expression transiently induces differentiation in preadipocytes and has an important role in inducing C/EBPα expression [27]. Both C/EBPβ and C/EBPδ accumulate to a maximal level during the first two days of differentiation and then decline sharply before the onset of C/EBPα accumulation [26]. Therefore, to examine the effects of **1** on the initial stage of adipocyte differentiation, mRNA levels of C/EBPβ and C/EBPδ were examined 48 h after adipocyte differentiation by quantitative real-time RT-PCR analysis (Figure 5C). Compound **1** significantly down-regulated C/EBPβ and C/EBPδ expression. In addition, **1** significantly blocked the induction of C/EBPα expression in differentiated adipocytes, as determined by western blot and quantitative real-time RT-PCR analysis (Figure 5A,B). These results suggest that **1** inhibits adipocyte differentiation, in part via inhibition of a C/EBPα dependent pathway.

The SREBP transcription factors regulate genes related to the metabolism of lipids and cholesterol [28]. Of the identified isomers of SREBP (SREBP1a, SREBP1c, and SREBP2), SREBP1c expresses lipogenic genes that encode enzymes involved in triglyceride synthesis and desaturation of fatty acids with a subsequent series of activations promoting the expression of FAS [29,30]. Down-regulation of SREBP1c by **1** might reduce fatty acid synthesis and may result in the inhibition of lipid accumulation by blocking adipogenesis. The inhibition of SREBP1c gene expression has been accompanied by a decrease in gene expression of SCD-1, ACC, and FAS, the target genes of SREBP1c. Highly expressed in adipose tissue, SCD-1 is a key rate-limiting enzyme in the desaturation of cellular lipids into monounsaturated fatty acids [31]. Cellular deprivation of the enzymatic activity of SCD-1 can lead to down-regulation of SREBP1, resulting in a decrease in lipogenesis [32]. The ACC are biotin-containing enzymes involved in fatty acid biosynthesis and oxidation; moreover, they catalyze the carboxylation of acetyl-CoA to malonyl-CoA, a rate-limiting step in fatty acid biosynthesis, through two enzymatic steps: biotin carboxylation and carboxyl group transfer [33]. ACC is mainly expressed in the cytosol of cells in lipogenic tissues, such as liver, adipose tissue, and lactating mammary gland and contributes to the *de novo* synthesis of fatty acids required for developmental processes [34]. FAS is a critical metabolic enzyme for lipogenesis that is highly expressed in liver and adipose tissue and that catalyzes the synthesis of saturated fatty acids in cells [35]. Therefore, a reduction in mRNA levels of SREBP1c and its target genes (SCD-1, ACC, and FAS) by treatment with **1** during adipocyte differentiation implies that inhibition of adipocyte differentiation can be mediated by the down-regulation of these lipogenic genes.

## 3. Experimental

### 3.1. Plant Materials

*S. pseudo-lasiogyne* twigs were collected from at the Medicinal Plant Garden, Seoul National University, Korea, in July 2009. Plant identification was authenticated by Dr. Jong Hee Park, Pusan National University, Korea. A voucher specimen (SNUPH-1105) has been deposited in the Herbarium of the Medicinal Plant Garden, College of Pharmacy, Seoul National University, Korea.

### 3.2. Extraction and Isolation

The dried plant material (17.0 kg) was pulverized and then extracted with 80% methanol (15 L, 3 h × 3) using ultrasonication at room temperature. The methanolic extract was concentrated *in vacuo* to result in a crude extract (1.21 kg). The crude extract was suspended in H_2_O and partitioned successively with *n*-hexane or EtOAc, resulting in solid residues of 48.0 g and 160.0 g, respectively, after removal of the solvents. Five salicortin derivatives were isolated from the EtOAc-soluble fraction and exhibited a significant inhibitory effect on adipocyte differentiation (17.7 ± 3.3% at 100 μg/mL). The derivatives were identified by spectral analysis as previously reported [14].

### 3.3. Cell Culture

Mouse embryo fibroblast 3T3-L1 cells were obtained from the American Type Culture Collection (Manassas, VA, USA) and incubated in Dulbecco’s modified Eagle’s medium (DMEM) supplemented with 10% bovine calf serum (BCS) until confluence. Two days after confluence (designated day 0), preadipocytes were stimulated to differentiate by adding differentiation medium (DM) containing DMEM with 10% fetal bovine serum (FBS), 0.5 mM 3-isobutyl-1-methylxanthine, 10 μg/mL insulin, and 1 μM dexamethasone for 2 d (days 0–2). During the subsequent two days (days 2–4), those cells were maintained in DM containing DMEM with 10% FBS and 10 μg/mL insulin. This was followed by an additional 4 d (days 4–8) of culture with DM containing DMEM with 10% FBS. All media contained 100 IU/mL penicillin and 100m g/mL streptomycin. Cells were maintained at 37 °C in a humidified atmosphere of 95% air and 5% CO_2_. The purity of each obtained compound was verified to be >98% by using high performance liquid chromatography. Test samples of the compounds were dissolved in dimethyl sulfoxide (DMSO) to a final concentration of 0.1% in media. Cell cultures were treated with test samples for the entire culture period (days 0–8).

### 3.4. Oil Red O staining

Lipid droplets in cells were stained with ORO. On day 8, culture dishes were washed three times with phosphate buffered saline (PBS) and fixed with 10% formalin for 1 h at room temperature. After fixation, cells were washed once with PBS and stained with a filtered ORO solution (6 parts saturated 0.6% ORO in isopropyl alcohol and 4 parts water) for 15 min at room temperature. Subsequently, cells were washed twice with water for 15 min then visualized. To quantify the intracellular lipids, spectrophotometric quantification of the stain was performed by dissolving the stained lipid droplets with 4% Nonidet P-40 in isopropyl alcohol for 5 min. Absorbance was measured at 544 nm.

### 3.5. Measurement of Cell Proliferation

For the MTT assay, cells were seeded in 96-well plates at a density of 5 × 10^3^ cells/well and incubated for 24 h. The 3T3-L1 cells were treated with vehicle or the compounds to be tested for 24 h and 48 h. The inhibitory activity of compounds on cell proliferation was assessed by MTT assay in which MTT (final concentration of 0.5 mg/mL in media) was directly added to cultures, followed by incubation at 37 °C for 4 h. Subsequently, the supernatant was aspirated and 100 μL of DMSO was added to dissolve the formazan. After the insoluble crystals were completely dissolved, absorbance at 540 nm was measured by using a microplate reader. Data were expressed as percentage cell viability relative to that in the control cultures.

### 3.6. Western Blot Analysis

The 3T3-L1 cells were washed twice with cold PBS and then lysed in an ice-cold modified RIPA buffer (Cell Signaling, Beverly, MA, USA) containing 0.5 mM dithiothreitol (DTT), 1 mM phenylmethanesulfonylfluoride (PMSF), and 1% protease inhibitor cocktail [36]. Total proteins (30 μg) were separated by 10% sodium dodecyl sulfate polyacrylamide gel electrophoresis and then transferred to polyvinylidene difluoride membranes (Millipore, Billerica, MA, USA). After blocking in tris-buffered saline and Tween 20 with 5% non-fat dry milk for 1 h at room temperature, the membrane was incubated overnight at 4 °C with 1:1000 diluted CCAAT/enhancer binding protein α (C/EBPα) and sterol regulatory element binding protein 1 (SREBP1c) primary antibodies (Santa Cruz Biotechnology, Santa Cruz, CA, USA). After incubation with 1:3000 diluted horseradish peroxidase-conjugated goat anti-rabbit immunoglobulin G secondary antibody (Santa Cruz Biotechnology) for 1 h at room temperature, immunoreactive proteins were visualized by adding an enhanced chemiluminescent solution (Amersham, Uppsala, Sweden). After normalization to β-actin, density values for the protein bands of interest were expressed as a percentage of the control by using ImageJ software (http://rsweb.nih.gov/ij).

### 3.7. Real-Time RT-PCR

Total RNA was extracted from the 3T3-L1 cells by using the RNease Plus Kit (QIAGEN Korea Ltd., Seoul, Korea). The cDNA was synthesized with 1 μg of total RNA by using the QuantiTech Reverse Transcription Kit (QIAGEN Korea), after which it was mixed with QuantiFast SYBR Green PCR master mix (QIAGEN Korea) along with specific primers in a total reaction volume of 20 μL. The PCR specific primers used with the QIAGEN kits for SYBR Green-based real-time RT-PCR were C/EBPα (NM_007678), C/EBPβ (NM_009883), C/EBPδ (NM_007679), SREBP1 (NM_011480), stearoyl coenzyme A desaturase 1 (SCD-1; NM_009127), fatty acid synthase (FAS; NM_007988), acetyl-CoA carboxylase (ACC; NM_133360), and glyceraldehyde 3-phosphate dehydrogenase (GAPDH; NM_008084) with all primers obtained from QIAGEN Korea*.* Amplification cycles were carried out at 95 °C for 20 s, 60 °C for 20 s, and 72 °C for 20 s. The last cycle was followed by a final extension step at 72 °C for 5 min. Quantitative SYBR Green real-time RT-PCR was performed by using an Applied Biosystems 7300 Real-Time PCR System (Life Technologies Corporation, Carlsbad, CA, USA) with the results analyzed by performing comparative C_t_ quantification [37]. GAPDH was amplified as an internal control. The C_t_ values of GAPDH were subtracted from the C_t_ values of the target genes (ΔC_t_). The ΔC_t_ values of the treated cells were compared with the ΔC_t_ values of the untreated cells.

### 3.8. Statistical Analysis

Statistical analysis was performed using Student’s *t* test. Data were expressed as mean ± standard deviation (SD). Statistical significance was represented with an asterisk for *p* values < 0.05, with two asterisks for *p* values < 0.01, and with three asterisks for *p* values < 0.001.

## 4. Conclusions

In conclusion, our study shows that an extract of *S*. *pseudo-lasiogyne* and its isolates, five salicortin derivatives, each bearing a 1-hydroxy-6-oxo-2-cyclohexenecarboxylate moiety, had anti-adipogenic activity in 3T3-L1 cells via suppression of adipocyte differentiation from preadipocytes. To investigate the inhibitory mechanism of salicortin derivatives on adipogensis in 3T3-L1 cells, the effects of 2′,6′-*O*-acetylsalicortin (**1**), the most potent of the five compounds assessed, on C/EBPα and SREBP1c expression levels were examined by western blot and quantitative PCR analyses. Treatment with **1** significantly down-regulated the expression of C/EBPα, C/EBPβ, C/EBPδ, SREBP1c, SCD-1, ACC, and FAS, affecting from early stage to terminal stage of adipogenesis. These results demonstrate that the anti-adipogenesis mechanism of **1** may be mediated via down-regulation of C/EBPα and SREBP1c dependent pathways. Therefore, salicortin derivatives may be potential novel therapeutic agents against obesity though their anti-adipogenic activities. Such activities should be assessed further through *in vivo* studies in animal models.
